# Iron status and fibroblast growth factor-23 in Gambian children

**DOI:** 10.1016/j.bone.2012.03.010

**Published:** 2012-06

**Authors:** Vickie Braithwaite, Landing M.A. Jarjou, Gail R. Goldberg, Ann Prentice

**Affiliations:** a Medical Research Council (MRC) Human Nutrition Research, Cambridge, UK; b MRC Keneba, West Kiang, The Gambia

**Keywords:** Iron deficiency, FGF23 metabolism, Gambian rickets

## Abstract

A relationship between iron and fibroblast growth factor-23 (FGF23) metabolic pathways has been proposed. Iron deficiency anaemia is prevalent in The Gambia and concentrations of fibroblast growth factor-23 FGF23 are elevated in a large percentage of Gambian children with rickets-like bone deformity.

We speculate that low iron status may be involved in the aetiology of Gambian rickets. The aim of this study was to determine if there was a relationship between haemoglobin, as a marker of iron status, and FGF23 in samples from children with and without a history of rickets-like bone deformities in The Gambia. We conducted a retrospective analysis of studies carried out from 2006 to 2008 in children from a rural community in The Gambia where iron deficiency anaemia is endemic and where elevated circulating concentrations of FGF23 have been found. To investigate the relationship between circulating FGF23 and haemoglobin concentrations we used an age-adjusted linear regression model on data from children < 18 y of age with a family or personal history of rickets-like bone deformity (BD) (*n* = 108) and from the local community (LC) (*n* = 382).

We found that circulating concentration of FGF23 was inversely correlated with haemoglobin concentration. This effect was more pronounced in BD children compared with LC children (interaction: *P ≤* 0.0001). Anaemia and elevated FGF23 were more prevalent in BD children compared to LC children (*P =* 0.0003 and *P =* 0.0001 respectively).

In conclusion, there is a stronger relationship between FGF23 and haemoglobin in Gambian children with a history of rickets compared to local community children. This study provides support for the contention that iron may be involved in FGF23 metabolic pathways.

## Highlights

► We analysed data from Gambian children with and without a family history of rickets. ► Hb is negatively correlated with FGF23. ► This relationship is significantly steeper in children with a history of rickets. ► Iron status may be involved in FGF23 metabolic pathways. ► Iron status may contribute to the aetiology of Gambian rickets.

## Introduction

A relationship between iron and FGF23 metabolic pathways has been proposed [1–4]. A study on a random selection of stored clinical biochemistry samples has indicated an inverse relationship between ferritin and FGF23 concentrations [3]. The authors hypothesised that these results may be highlighting the role of iron in the processing and excretion of the FGF23 molecule in the kidney [3]. In addition, a study of autosomal dominant hypophosphataemic rickets (ADHR) patients and controls indicated a negative relationship between serum iron and FGF23 concentrations [4]. Furthermore a study of mice with ADHR has shown that a diet low in iron can induce elevated FGF23 concentrations [5].

Studies in children in The Gambia, West Africa have shown that anaemia is endemic and that iron deficiency is the predominant cause of anaemia throughout the year [6]. A national survey conducted in 2001 indicated that 76% of Gambian children under the age of 5 y had anaemia, defined as having haemoglobin (Hb) < 11.0 g/dl [7]. In addition, cases of non-vitamin D deficiency rickets have been reported in Gambian children with chronically elevated circulating FGF23 concentrations [8]. It has been proposed that a chronically low dietary calcium supply resulting in a 1,25-dihydroxyvitamin D (1,25(OH)_2_D)-driven increase in FGF23 concentration and consequent excessive urinary phosphate loss may be contributing to the aetiology of Gambian rickets [8,9].

To investigate the possible link between iron status and FGF23 concentration a post-hoc analysis was conducted on existing data from previous studies on Gambian children both with and without a family or personal history of rickets-like bone deformities. Hb was used as the only available marker of iron status and data collection was conducted predominantly outside of the malaria season. The aims of this analysis were to identify any relationship between circulating concentrations of Hb and FGF23, to identify any differences in this relationship between Gambian children with and without a history of rickets-like bone deformities and to consider if iron may be involved in FGF23 metabolic pathways.

## Materials and methods

### Patients and study design

Existing data were obtained from three studies conducted previously at MRC Keneba, The Gambia. Written informed consent was obtained from parents of children involved in the three studies. Ethical approval for the original studies and the analysis of existing data was given by The Gambian Government/MRC Laboratories Joint Ethics Committee. Data from children under the age of 18.0 y with no acute illness a week prior to the study and with measurements for both FGF23 and Hb were included.

#### Children with a personal or family history of rickets-like bone deformity (BD children)

Data from 32 of the 35 children with a history of rickets-like bone deformities (BD Index) as described in [9] and their siblings (*n* = 76) (BD Siblings) were obtained from an aetiological follow-up study of rickets in The Gambia and were selected on the basis of fitting the inclusion criteria (see Patients and study design section). Measurements of these children were made between May–September 2006. At presentation the BD Index children were characterised by 25-hydroxyvitamin D (25OHD) concentration in the normal range, elevated FGF23 and 1,25(OH)_2_D concentrations and a low plasma phosphate (P) concentration. The elevations in FGF23 were observed over a year of treatment with calcium (Ca) and vitamin D as described in [8]. At follow-up at a mean of 4 y, 16 of the BD Index children included in these analyses had lasting leg deformities [9].

#### Children from the local community (LC children)

Data were obtained from two community studies to provide anthropometry and biochemistry from outwardly healthy children (LC children) (*n* = 382) who were selected on the basis of fitting the inclusion criteria (see Patients and study design section). The protocol for the first study (*n* = 74) has been described elsewhere [9]. The children were measured in January–February (*n* = 26) and September–October 2007 (*n* = 48)*.* The second study was a follow-up (Jarjou LMA, and Prentice A, unpublished) of children (*n* = 308) born to mothers who had previously participated in a Ca supplementation study during pregnancy (ISRCTN96502494), and who had previously taken part in a study of blood pressure at ages 5–10 y [10]. These data were collected from May–October 2007 and April–August 2008.

#### Anthropometry

Weight was measured to the nearest 0.1 kg using a calibrated electronic scale (model HD-314, Tanita B.V., Hoofddorp, The Netherlands). Height was measured to the nearest mm using a portable stadiometer (Leicester Height Measure, SECA, Hamburg, Germany). Sitting height was also measured in BD children to the nearest mm using the same portable stadiometer. Body mass index (BMI) was calculated by dividing weight (kg) by height^2^ (m^2^).

#### Fasting blood and 2 h urine collection

An overnight-fasted, 2 h urine sample was collected between the hours of 0700–0900. Acidified (HCl 10 μl/ml, laboratory reagent grade SD 1.18, Fisher Scientific) urine aliquots were stored at − 20 °C and then later transported frozen on dry ice to MRC HNR, Cambridge, UK where they were stored at − 20 °C until analysis.

A fasting, antecubital venous blood sample (5–15 ml according to the age of the child) was collected 1 h after the start of the 2 h urine collection and was transferred to pre-cooled lithium–heparin (LiHep) and ethylenediaminetetraacetic acid (EDTA)-coated tubes. Blood ionised Ca (*i*Ca) and Hb were measured in whole blood (ABL77, Radiometer Medical, MA, USA) within 10 min, and pH 7.4 corrected values for *i*Ca were used. The remainder of the blood was separated by centrifugation at 4 °C within 45 min and frozen at − 70 °C, and later transported frozen on dry ice to MRC HNR where it was stored at − 80 °C until analysis.

#### Biochemical analysis

The samples were analysed for markers of vitamin D, Ca and P metabolism and of renal function, using commercially-available methods according to the manufacturers' instructions. EDTA-plasma was used for the analysis of intact parathyroid hormone (PTH) and C-terminal FGF23; LiHep-plasma was used for other analyses. PTH was measured by immunoradiometric assay (DiaSorin Ltd, UK) and FGF23 was analysed using a 2nd generation C-terminal, two-site enzyme-linked immunosorbant assay (Immutopics Inc.,CA, USA). For FGF23 the manufacturer's upper limit of the reference range of 125 RU/ml was used as a cut-off of normality and > 1000 RU/ml was considered grossly elevated. Plasma 25OHD and 1,25(OH)_2_D were measured by radioimmunoassay (DiaSorin, Stillwater, MN, USA and IDS, UK respectively). For 25OHD, < 25 nmol/l was taken as an indicator of increased risk of vitamin D-deficiency rickets [11]. The following colorimetric methods (Koni Analyser 20i, Finland) were used to determine plasma analytes: P, ammonium molybdate; albumin, bromocresol purple; total alkaline phosphatase (TALP), p-nitrophenol and cystatin C (Cys C), immunoprecipitation. Acidified urine was used to determine urinary (*u)* P, Ca and creatinine (Cr) employing the same colorimetric methods as for plasma P, the arsenazo III method for *u*Ca and the Jaffe method for *u*Cr. Standards used in *u*rinary assays were acidified prior to use. Assay accuracy and precision were monitored across the working range of the assays using reference materials provided by external quality assurance schemes (National-external-quality-assessment-scheme (NEQAS), Department of Clinical Biochemistry, Royal Infirmary, Edinburgh, UK: Vitamin D-external-quality-assessment-scheme (DEQAS), Endocrine/Oncology Laboratory, Charing Cross Hospital, London, UK) or purchased commercially (Radiometer Medical, MA, USA and Roche Human Control, Roche Diagnostics Ltd, UK) and kit controls supplied by the manufacturer. In addition, an aliquot of a pooled plasma sample was assayed in each batch to monitor possible drift over time and to provide running quality assurance for analytes where no external reference material was available.

#### Statistical analysis and calculations

Multiple regression tests were performed using DataDesk 6.1 (Data Description Inc., NY, USA) and two-tailed Chi-square tests (without Yates' correction) were performed using GraphPad QuickCalcs (GraphPad Software, Inc.). Normally distributed data are presented as mean (1SD), positively skewed distributions of data are presented as geometric mean (− 1SD, + 1SD) obtained from the antilog of mean (1SD) for the logged values. Variables with positively skewed distributions were transformed to natural logarithms before further statistical analysis.

Regression analysis was used to assess the relationships between age (as a continuous variable), group, and sex with each variable (anthropometric or biochemical). To determine differences in the relationships between variables and group (BD Index *vs.* BD Sibling, BD *vs.* LC, anaemic *vs.* non-anaemic or FGF23 > 125 RU/ml *vs.*FGF23 ≤ 125 RU/ml) an interaction term (group × independent variable) was used in the model as an independent variable. Sex was not found to be a significant factor in predicting any of the variables, and therefore was not included in the models presented in this paper. However, weight, height, BMI, 25OHD, *i*Ca, P, TALP, Hb, FGF23, 1,25(OH)_2_D, PTH, *u*P:*u*Cr and tubular maximal reabsorption of phosphate (TmP:GFR) were influenced by age. Age, therefore, was added as an independent variable in regression models and age-adjusted data have been used throughout the text and in the tables. In addition, age-adjusted FGF23 and Hb values were calculated to explore the relationship further. Age-adjustment for Hb was derived by including log_e_Hb and age in the regression model separately for each group (BD or LC); evaluating the residual for each subject; adding the residual to log_e_ (mean group Hb) value; and calculating the antilog. Age-adjusted FGF23 was derived using the same method.

Children were defined as being anaemic based on Hb thresholds from UK Scientific Advisory Committee on Nutrition (SACN) guidelines: 5–11.99 y ≤ 11.5 g/dl, 12–14.99 y (and non-pregnant females > 15 y) ≤ 12.0 g/dl, and males > 15 y ≤ 13.0 g/dl [12]. No seasonal differences were seen in the FGF23 or Hb measurements and therefore season was not incorporated into any analyses.

Estimated glomerular filtration rate (eGFR) ml/min, was derived by eGFR = [74.835/(Cys C(mg/l)^1/0.75^)] ml/min [13]. TmP:GFR (mmol/l) was determined in the following way: tubular reabsorption of phosphate (TRP) = 1 − {(*u*P/P) × (Cr/*u*Cr)}, if TRP < 0.86 then TmP:GFR = TRP × P mmol/l, if TRP > 0.86 then TmP:GFR = (0.3 × TRP / {1 − (0.8 × TRP)}) × P mmol/l [14]. *u*P and *u*Ca were expressed as a molar ratio with *u*Cr (*u*P:*u*Cr and *u*Ca:*u*Cr respectively).

## Results

### Characterisation of BD and LC children

The children as a whole (*n* = 490) had a mean age of 8.9 (3.0) y and 51% were female. When looking at the children with a personal or a family history of rickets-like bone deformities (BD) there was no difference between Index children (*n* = 32) or their siblings (*n* = 76) in any variables before and after age-adjustments were made, with the exception of height where the BD siblings tended to be taller than the index children (*P* = 0.03) (data not shown.). There was no significant difference in age or sex ratio between BD children (*n* = 108) and the children from the local community (LC) (*n* = 382) (Table 1). The children from both groups were not significantly different in height but the BD children were heavier and had a greater BMI compared to LC children after adjusting for age (*P* ≤ 0.0001 and *P* ≤ 0.0001 respectively). This difference was unlikely to be fully accounted for by the lasting leg deformities in some of the BD Index children; there was a strong correlation between sitting and standing height (R^2^ = 98.0%). In addition the difference between BMI in BD and LC remained when BD Index children with lasting leg deformities were excluded (*P* ≤ 0.0001).

#### Biochemical profile of BD and LC children

All of the children, with the exception of *n* = 2 LC children, had a plasma 25OHD concentration above 25 nmol/l but there was no significant difference in mean 25OHD concentration between BD and LC children. BD children had higher 1,25(OH)_2_D, and lower Hb than LC children (*P* ≤ 0.0001 and *P =* 0.0006 respectively). *u*P:*u*Cr, and *u*Ca:*u*Cr were higher, and TmP:GFR was lower in BD children than in LC children (*P* ≤ 0.0001, *P* = 0.009, and *P =* 0.0007 respectively). Cys C tended to be higher and eGFR was lower in BD children than in LC (*P =* 0.02 and *P =* 0.03 respectively). Albumin was higher in BD children than in LC children (*P =* 0.01) and there was no significant difference in plasma *i*Ca and P concentration between the groups (Table 1).

#### Anaemia

The prevalence of anaemia was higher in BD children compared with LC children (15% *vs.* 6%, *χ*^2^ = 8.2, *P =* 0.0004). Anaemic children were younger than non-anaemic children (*P =* 0.006). After adjusting for age, anaemic children (*n* = 40) tended to be shorter, and heavier and had a greater BMI than non-anaemic children (*n* = 450) (*P =* 0.02, *P* = 0.02 and *P* = 0.006 respectively) (Table 2). Plasma FGF23 and 1,25(OH)_2_D concentrations were higher in children with anaemia compared to those without (*P* ≤ 0.0001 and *P =* 0.03 respectively). There was no significant difference in 25OHD or PTH between the two groups but *i*Ca was higher in the anaemic children (*P =* 0.007). TmP:GFR tended to be lower and *u*Ca:*u*Cr was higher in anaemic children compared to non-anaemic children (*P =* 0.04 and *P* = 0.0003 respectively) but there was no difference in eGFR or in plasma P. Albumin was lower in anaemic children compared to those without (*P* ≤ 0.0001).

#### Predictors of elevated FGF23 concentrations

27% of BD children (*n* = 29) had circulating concentrations of FGF23 above the upper limit of normal (> 125 RU/ml) compared to 13% of LC children (*n* = 48) (*χ*^2^ = 12.9, *P =* 0.0003). 8% of BD children (*n* = 9) had grossly elevated concentrations (> 1000 RU/ml) compared with 2% of LC children (*n* = 2) (*χ*^2^ = 11.3, *P =* 0.0008). There was no difference in the number of BD Index or BD Sibling children with concentrations of FGF23 > 125 or > 1000 RU/ml (*P* = 0.1 and *P* = 0.2 respectively). Children with high FGF23 were younger than children with FGF23 within the normal range (*P =* 0.0001) independent of group. After adjusting for age, all children with high FGF23 (> 125 RU/ml) were shorter, tended to be heavier and had a greater BMI than children with FGF23 concentrations within the normal range (*P* ≤ 0.0001, *P =* 0.03 and *P* ≤ 0.0001 respectively) (Table 3). 1,25(OH)_2_D and Cys C were higher in children with high FGF23 (*P =* 0.0002 and *P =* 0.02 respectively) and Hb was lower (*P* ≤ 0.0001). eGFR and TmP:GFR were lower, and *u*P:*u*Cr and *u*Ca:*u*Cr were higher in children with high FGF23 concentrations compared to those with FGF23 within the normal range (*P =* 0.02, *P =* 0.05, *P* = 0.02 and *P* = 0.02 respectively). There was no significant difference in *i*Ca, 25OHD, PTH, P, TALP or albumin between the two groups.

In univariate regression models the dependent variable log_e_FGF23 was negatively associated with log_e_Hb, log_e_TALP, log_e_eGFR, height and weight (log_e_Hb: coefficient = − 2.54(SE 0.39), t-ratio = − 6.41, *P* ≤ 0.0001, R^2^ = 7.6%; log_e_TALP: coefficient = − 0.47(SE 0.15), t-ratio = 3.09, *P* = 0.002, R^2^ = 1.7%; log_e_eGFR: coefficient = − 0.46(SE 0.21), t-ratio = − 2.15, *P* = 0.03, R^2^ = 0.7%, height: coefficient = − 2.08(SE 0.21), t-ratio = − 9.58, *P* ≤ 0.0001, R^2^ = 15.7%; and weight: coefficient = − 0.03(SE 0.004), t-ratio = − 6.51, *P* ≤ 0.0001,R^2^ = 7.9%) and positively associated with log_e_1,25(OH)_2_D, cystatin C and log_e_*u*P:*u*Cr (log_e_1,25(OH)_2_D: coefficient = 0.52(SE 0.12), t-ratio = 4.14, *P* ≤ 0.0001, R^2^ = 3.2%; cystatin C: coefficient = 0.78(SE 0.35), t-ratio = 2.25, *P* = 0.02, R^2^ = 0.8%; and log_e_*u*P:*u*Cr: coefficient = 0.23(SE 0.06), t-ratio = 3.66, *P* = 0.0003, R^2^ = 2.5%). In a multivariate regression model with the dependent variable log_e_FGF23 against all of the significant independent variables from univariate analysis log_e_Hb and height were the two strongest predictors of log_e_FGF23.

#### FGF23 and haemoglobin

**Fig. 1:**
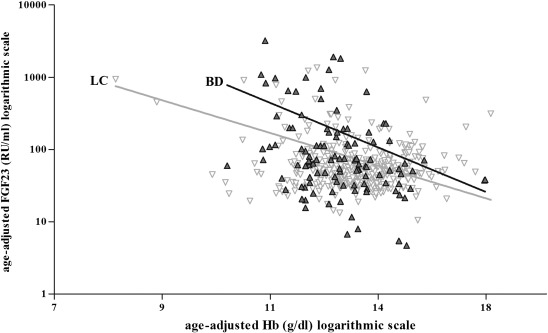
Relationship between age-adjusted haemoglobin (Hb) (g/dl) and age-adjusted FGF23 (RU/ml) (on a logarithmic scale) grouped into BD children (bone deformity) ▴ and LC children (local community) ▿. Age-adjusted Hb was derived by including log_e_ Hb and age in the regression model separately for each group (BD or LC); evaluating the residual for each subject; adding the residual to log_e_ (mean group Hb) value; and calculating the antilogarithm. Age-adjusted FGF23 was derived using the same method. The equation of the line derived from multiple regression using unadjusted log_e_ FGF23 (y) with unadjusted log_e_Hb and age (x) for: BD children only was log_e_FGF23 = [16.05 (SE 3.16)] − [4.28 (SE 1.27) (log_e_Hb)] − [0.08 (SE 0.03) (age)], R2 = 23.2%, *P =* 0.001 and LC children only was log_e_FGF23 = [7.69 (SE 0.95)] − [1.08 (SE 0.38) (log_e_Hb)] − [0.08 (SE 0.01) (age)], R^2^ = 10.4%, *P =* 0.005. All) both BD and LC children together was log_e_FGF23 = [9.59 (SE 0.99)] − [1.77 (SE (0.40) (log_e_Hb)] − [0.09 (SE 0.01) (age)], R^2^ = 15.5%, *P* ≤ 0.0001 (line not shown). The log_e_Hb × group interaction was highly significant (*P* < 0.0007) demonstrating differences between BD and LC in the slopes of the relationship.

Hb was a strong independent negative predictor of FGF23 concentration after adjusting for age; the coefficient for log_e_Hb = − 1.77(SE 0.40), t-ratio = − 4.48, *P* ≤ 0.0001 (Fig. 1). This effect, however, was more pronounced in BD children (coefficient = − 4.28 (SE 1.27), t-ratio = − 3.37, *P =* 0.001) compared to LC children (coefficient = − 1.08 (SE 0.38), t-ratio = − 2.84, *P =* 0.005) (Fig. 1). Furthermore the age-adjusted relationship between FGF23 and Hb was different in BD and LC children (test for interaction *P* = 0.0007). When excluding the two LC children with Hb concentrations lower than 9 g/dl, the age-adjusted relationship between FGF23 and Hb in LC children was no longer present (*P =* 0.2). However, the group interaction term (BD *vs.*LC) was still significant (*P* ≤ 0.0001). There was no significant difference in the relationship between Hb and FGF23 in BD Index and BD Sibling children (*P* = 0.01 and *P* = 0.03 respectively, test for interaction: *P* = 0.5); BD Index log_e_FGF23 = [18.65(SE 5.6)] − [5.82(SE 2.21)(log_e_Hb)] − [0.04(SE 0.09)(age)] and BD Sibling log_e_FGF23 = [14.3(SE 3.82)] − [3.47(SE 1.54)(log_e_Hb)] − [0.10(SE 0.03)(age)]. The FGF23 *vs*. Hb correlation and the significant group interaction were not materially different in multiple regression models that also included weight, height and albumin to account for any confounding due to differences in nutritional status. In these models weight and height were significant predictors of FGF23 in addition to Hb (positive and negative respectively), but age and albumin were not (data not shown).

## Discussion

This study has demonstrated an inverse relationship between Hb and FGF23 concentrations which is in keeping with other reports suggesting a link between iron status and FGF23 metabolism. These include Durham et al. with ferritin and FGF23 concentrations [3], Imel et al. with serum iron and FGF23 concentrations [4] and Farrow et al. showing that a diet low in iron can induce elevated FGF23 concentrations in an ADHR mouse model [5]. The inverse relationship between Hb and FGFG23 was apparent when the data were examined as a whole but the magnitude of the negative slope was significantly different between BD and LC children, being steeper in the BD children. Once the more severely anaemic LC children were excluded there was no longer a significant relationship between Hb and FGF23 in LC children; however, the group difference in the relationship remained.

To our knowledge, the data from LC children represent one of the largest sets of FGF23 concentrations from a healthy, although generally undernourished, paediatric population living in a low-income country. One of the interesting findings from this study was that FGF23 was not only elevated in children with a personal or family history of rickets-like bone deformities but also, albeit to a lesser extent, in some apparently healthy children living in the local community. 13% of LC children had FGF23 concentrations over the upper limit of normal (> 125 RU/ml) compared with 27% of BD children. Furthermore 2% of LC children had FGF23 concentrations over 1000 RU/ml, which are concentrations generally only reported in patients with clinical pathologies such as hereditary hypophosphatemic rickets and chronic kidney disease [15]. Another interesting finding is that unaffected siblings of children with a history of rickets-like bone deformities had biochemical profiles more similar to their affected siblings than to children from the local community. This suggests genetic factors and/or the household environment may be contributing to these results.

One of the consistent results in this study and our previous studies [9] is a possible involvement of the kidney in the aetiology of Gambian rickets. The BD and LC children with elevated FGF23 have lower eGFR albeit within the normal range. In addition the BD children were shorter, heavier and had a higher BMI than LC children. This finding remained even after the BD Index children with lasting leg deformities were excluded.

The C-terminal ELISA kit (Immutopics) was used to determine the circulating concentrations of FGF23. This assay can detect both the biologically active, intact FGF23 hormone and the biologically inactive C-terminal FGF23 fragment [16]. Researchers have hypothesised that iron may act on FGF23 pathways in the following ways; firstly by inhibiting the cleavage of the intact FGF23 molecule and secondly in assisting the clearance of FGF23 fragments by the kidney [3]. It is possible that a low eGFR could result in an accumulation of C-terminal FGF23 fragments and would thus contribute to a greater amount of FGF23 detected by the assay. However, the lower TmP:GFR in BD children and, therefore greater urinary phosphate excretion, indicates the presence of biologically active and intact FGF23. Thus the FGF23 that we have detected is likely to be predominantly the biologically functional, intact FGF23 molecule which is decreasing phosphate reabsorption in the renal tubules. However, despite a higher FGF23 concentration and associated greater urinary phosphate excretion, the BD children showed no signs of hypophosphatemia. The ability of Gambian children, in general, to maintain normophosphatemia in the face of an elevated FGF23 concentration may be explained by the low Ca-to-P ratio of the Gambian diet which would be expected to result in enhanced intestinal absorption of P, as we have described elsewhere [9].

Iron deficiency and malaria are the two major causes of anaemia in The Gambia [6,17]. Iron deficiency is the predominant cause for anaemia throughout the year [6] whereas the majority of malarial anaemia is seen during the months of September–December [18]. 84% of all children in this study and 68% of anaemic children were measured outside of September–October. Only *n* = 2 BD children were measured during the malaria season. Although we cannot discount the possibility that Hb was marking underlying traits such as sickle-cell anaemia or thalassemia, the results from this study therefore suggest that, although direct markers of iron status were not measured, it is likely that Hb concentration was an indicator of iron status in more than 70% of children with anaemia.

Therefore, in conclusion this study supports the contention that iron is involved in FGF23 metabolic pathways. Furthermore it has shown that this effect is more pronounced in children with a personal or family history of rickets-like bone deformities. It has been proposed that rickets in The Gambia is predominantly caused by a chronically low dietary calcium supply leading to increased FGF23, and associated urinary phosphate loss [8,9]. It is possible that poor iron status may also play a role in the elevation of FGF23 concentrations and therefore may be a contributing factor to Gambian rickets.

## Figures and Tables

**Table 1:** Data from children with a personal or family history of rickets-like bone deformities (BD) and children from the local community (LC).

Dependent	BD children	LC children	Difference	*P-value*
*Variable*	*n* = 108	*n* = 382	*Age-adjusted*	

*Characteristics*				

Age (y)	8.7 (4.5)	8.9 (2.4)	− 0.3	0.4
Sex (F/M)	52/56	199/183	–	0.5
Height (m)	1.23 (0.3)	1.26 (0.1)	− 0.01	0.14
Weight (kg)	26.5 (13.7)	23.6 (6.8)	3.3	0.0001
BMI (kg/m^2^)	16.0 (2.5)	14.6 (1.5)	1.4	0.0001


*Biochemistry*				

*i*Ca (7.4) (mmol/l)	1.13 (0.05)	1.13 (0.05)	− 1.8x10^− 8^	0.9
25OHD (nmol/l)	62.5 (14.7)	61.8 (15.8)	0.6	0.7
1,25(OH)_2_D* (pmol/l)	262.9 (184.5, 374.5)	217.3 (160.9, 293.3)	0.2*	0.0001
PTH* (pg/ml)	49.9 (28.6, 87.2)	54.7 (34.3, 87.4)	− 1.2*	0.2
P (mmol/l)	1.51 (0.22)	1.48 (0.18)	0.2	0.3
FGF23* (RU/ml)	77.9 (19.5, 310.8)	65.5 (30.9, 138.4)	0.1*	0.12
TALP* (U/l)	293 (204, 421)	291 (229, 368)	0.005*	0.9
Cys C (mg/l)	0.83 (0.12)	0.80 (0.12)	0.03	0.02
Hb* (g/dl)	12.8 (11.6, 14.1)	13.3 (12.0, 14.7)	− 0.04*	0.0006
Albumin (g/l)	39.5 (3.6)	38.7 (2.9)	0.8	0.01


*eGFR and mineral excretion*				

eGFR* (ml/min)	96.6 (79.9, 117.0)	101.4 (83.5, 123.1)	− 0.05*	0.03
TmP:GFR (mmol/l)	1.67 (0.32)	1.77 (0.26)	− 0.1	0.0007
*u*P:uCr*	1.69 (0.97, 2.95)	1.20 (0.64, 2.26)	0.33*	0.0001
*u*Ca:uCr*	0.07 (0.02, 0.26)	0.05 (0.01, 0.18)	0.37*	0.009

For normally distributed data, the results are mean (SD); for positively skewed data (denoted by *) the results are geometric mean (− 1SD, + SD). The age-adjusted difference and *P*-value (BD children = 1, LC children = 0) were determined by regression analysis or from chi-square (for sex); age and sex were un-adjusted. The difference is expressed in original units for normally distributed data and as a proportion for skewed data.

**Table 2:** Data from children with and without a personal or family history of rickets-like bone deformities (BD and LC) with and without anaemia.

Dependent	Anaemic	Non-anaemic	Difference	*P-value*
*Variable*	*n* = 40	*n* = 450	*Age-adjusted*	

*Characteristics*				

Age (y)	7.66 (4.31)	9.0 (2.83)	− 1.3	0.006
Sex (F/M)	21/19	230/220	–	0.9
Height (m)	1.16 (0.26)	1.26 (0.16)	− 0.02	0.02
Weight (kg)	22.5 (13.7)	24.4 (8.32)	1.7	0.02
BMI (kg/m^2^)	15.3 (2.4)	14.9 (1.8)	0.8	0.006


*Biochemistry*				

*i*Ca (7.4) (mmol/l)	1.15 (0.04)	1.26 (0.05)	0.02	0.007
25OHD (nmol/l)	65.1 (19.9)	61.7 (15.1)	2.6	0.3
1,25(OH)_2_D* (pmol/l)	256.7 (182.2, 361.5)	224.0 (162.9, 308.1)	0.1*	0.03
PTH* (pg/ml)	53.9 (37.2, 78.0)	53.8 (32.7, 88.4)	0.6*	0.6
P (mmol/l)	1.46 (0.20)	1.49 (0.19)	− 0.05	0.08
FGF23* (RU/ml)	157.2 (32.6, 758.9)	63.2 (28.1, 141.8)	0.8*	0.0001
TALP* (U/l)	270.4 (187.8, 389.3)	293.1 (226.4, 379.4)	− 0.09*	0.02
Cys C (mg/l)	0.81 (0.12)	0.81 (0.11)	0.01	0.5
Hb* (g/dl)	10.8 (9.9, 11.8)	13.4 (12.4, 14.5)	− 0.2*	0.0001
Albumin (g/l)	36.9 (4.1)	39.1 (2.9)	− 2.1	0.0001


*eGFR and mineral excretion*				

eGFR* (ml/min)	98.6 (78.7, 123.4)	100.5 (83.0, 121.7)	− 0.01*	0.7
TmP:GFR (mmol/l)	1.68 (0.31)	1.76 (0.3)	− 0.09	0.04
*u*P:uCr*	1.61 (0.99, 2.61)	1.27 (0.67, 2.40)	0.17*	0.10
*u*Ca:uCr*	0.11 (0.04, 0.34)	0.05 (0.01, 0.18)	0.77*	0.0003

For normally distributed data, the results are mean (SD); for positively skewed data (denoted by *) the results are geometric mean (− 1SD, + SD). The age-adjusted difference and *P*-value (anaemic = 1, non-anaemic = 0) were determined by regression analysis or from chi-square (for sex); with the exception of age and sex which were un-adjusted. The difference is expressed in original units for normally distributed data and in proportion for skewed data.

**Table 3:** Data from children with and without a personal or family history of rickets-like bone deformities (BD and LC) divided by FGF23 concentration.

Dependent	FGF23 > 125	FGF23 ≤ 125	Difference	*P-value*
*Variable*	*n* = 77	*n* = 413	*Age-adjusted*	

*Characteristics*				

Age (y)	6.65 (3.69)	9.32 (2.66)	− 2.7	0.0001
Sex (F/M)	43/34	208/205	–	0.4
Height (m)	1.10 (0.24)	1.28 (0.14)	− 0.04	0.0001
Weight (kg)	19.4 (9.7)	25.2 (8.4)	1.3	0.03
BMI (kg/m^2^)	15.0 (1.6)	14.9 (1.9)	0.9	0.0001


*Biochemistry*				

*i*Ca (7.4) (mmol/l)	1.13 (0.05)	1.13 (0.05)	0.0005	0.4
25OHD (nmol/l)	62.7 (17.2)	61.8 (15.3)	− 0.9	0.6
1,25(OH)_2_D* (pmol/l)	264 (192, 362)	220.2 (160, 303)	0.1	0.0002
PTH* (pg/ml)	53.6 (30.7, 93.3)	53.8 (33.5, 86.6)	1.3*	0.2
P (mmol/l)	1.52 (0.23)	1.48 (0.18)	− 0.009	0.7
FGF23* (RU/ml)	379.3 (152.6, 942.4)	49.4 (31.1, 78.4)	1.9*	0.0001
TALP* (U/l)	304 (224, 414)	289 (223, 375)	0.02*	0.6
Cys C (mg/l)	0.84 (0.13)	0.80 (0.11)	0.04	0.02
Hb* (g/dl)	12.3 (10.8, 14.0)	13.3 (12.2, 14.6)	− 0.06*	0.0001
Albumin (g/l)	38.7 (3.4)	38.9 (3.1)	− 0.4	0.4


*eGFR and mineral excretion*				

eGFR* (ml/min)	95.2 (77.8, 116.4)	101.4 (83.7, 122.7)	− 0.06*	0.02
TmP:GFR (mmol/l)	1.72 (0.35)	1.76 (0.26)	− 0.07	0.05
*u*P:uCr8*	1.66 (0.85, 3.24)	1.24 (0.67, 2.28)	0.19*	0.02
*u*Ca:uCr*	0.08 (0.02, 0.33)	0.05 (0.02, 0.18)	0.4*	0.02

For normally distributed data, the results are mean (SD); for positively skewed data (denoted by *) the results are geometric mean (− 1SD, + SD). The age-adjusted difference and *P*-value (FGF23 > 125 = 1 and FGF23 ≤ 125 = 0) were determined by regression analysis or from chi-square (for sex); with the exception of age and sex which were un-adjusted. The difference is expressed in original units for normally distributed data and in proportion for skewed data. NB. When adjusted for age the children with FGF23 > 125 were significantly heavier than FGF23 **≤** 125 which is why the difference is + 0.03.
